# Multidisciplinary patient‐centered program implemented in a psychogeriatric unit in Brazil:Caregiver Burden Outcomes

**DOI:** 10.1002/alz.093213

**Published:** 2025-01-09

**Authors:** Patricia Cardoso Buchain, Debora Pastore Bassitt, Tania Correa de Toledo Ferraz Alves

**Affiliations:** ^1^ Department & Institute of Psychiatry Faculty of Medicine ‐ University of São Paulo, São Paulo Brazil; ^2^ Institute of Psychiatry USP Medical School, Brazil, São Paulo Brazil; ^3^ Institute of Psychiatry, University of Sao Paulo, Brazil, Sao Paulo Brazil

## Abstract

**Background:**

Caregiver burden is the result of dealing with the dependency of the subject who needs attention and care. Caregivers of people with neuropsychiatric disease could experience emotional, physical and financial stress. The literature shows that the caregiver burden can negatively impact both, the caregiver and the patient. According to evidence, psychosocial interventions may decrease BPSD, depressive symptoms and caregiver burden. At Institute of Psychiatry USP Medical School, Brazil we offer psychosocial interventions by a multidisciplinary team in a transitional care service (day‐hospital unit). In this report we will analyse the impact of this intervention in caregiver burden.

**Method:**

The program involves interventions at day‐hospital offering management of psychoactive drug treatment, prevention and care of geriatric neuropsychiatric syndromes, and support to family caregivers, for approximately two months. There are groups and individual interventions lead by a multidisciplinary team: medical team, nurse, psychologist, speech therapist, physiotherapist, occupational therapist, nutritionist and social worker. After an initial assessment an individualised care plan is established and also the caregivers are involved in the focus of intervention. The care related to caregivers involves psychoeducation, organising routine, strategies to manage BPSD and emotional support. To assess caregiver burden we use the Zarit scale. The intervention to caregivers involves psychoeducation groups (60‐90 min), and individual sessions.

**Result:**

There were 30 consecutive admissions between march and december 2023, the main diagnosis at admission were depression and dementia. The mean age of the patients 71,24, gender 19 female, 11 male. Admission mean Zarit was 36,23 and discharged 28,84.

**Conclusion:**

The therapeutic program demonstrates efficiently to caregivers in order to adapt to behaviour changes and develop a successful daily routine care at home and decreased caregiver burden. Our findings demonstrated that this patient‐centered program results in reducing burden from /moderate to severe/ to /mild to moderate/. The present intervention is an important strategy to assist caregivers.

